# Chloroquine Interference with Hemoglobin Endocytic Trafficking Suppresses Adaptive Heme and Iron Homeostasis in Macrophages: The Paradox of an Antimalarial Agent

**DOI:** 10.1155/2013/870472

**Published:** 2013-06-11

**Authors:** Christian A. Schaer, Endre Laczko, Gabriele Schoedon, Dominik J. Schaer, Florence Vallelian

**Affiliations:** ^1^Department of Anesthesiology, University Hospital, 8091 Zurich, Switzerland; ^2^Functional Genomics Center, University of Zurich, 8057 Zurich, Switzerland; ^3^Division of Internal Medicine, University Hospital, 8091 Zurich, Switzerland

## Abstract

The CD163 scavenger receptor pathway for Hb:Hp complexes is an essential mechanism of protection against the toxicity of extracellular hemoglobin (Hb), which can accumulate in the vasculature and within tissues during hemolysis. Chloroquine is a lysosomotropic agent, which has been extensively used as an antimalarial drug in the past, before parasite resistance started to limit its efficacy in most parts of the world. More recent use of chloroquine is related to its immunomodulatory activity in patients with autoimmune diseases, which may also involve hemolytic disease components. In this study we examined the effects of chloroquine on the human Hb clearance pathway. For this purpose we developed a new mass-spectrometry-based method to specifically quantify intracellular Hb peptides within the endosomal-lysosomal compartment by single reaction monitoring (SRM). We found that chloroquine exposure impairs trafficking of Hb:Hp complexes through the endosomal-lysosomal compartment after internalization by CD163. Relative quantification of intracellular Hb peptides by SRM confirmed that chloroquine blocked cellular Hb:Hp catabolism. This effect suppressed the cellular heme-oxygenase-1 (HO-1) response and shifted macrophage iron homeostasis towards inappropriately high expression of the transferrin receptor with concurrent inhibition of ferroportin expression. A functional deficiency of Hb detoxification and heme-iron recycling may therefore be an adverse consequence of chloroquine treatment during hemolysis.

## 1. Introduction 

Extracellular hemoglobin (Hb) is the pathophysiologic consequence of hemolysis and is not innocuous [1]. The injurious impact of free Hb has been ascribed to heme-driven oxidative processes and vascular dysfunction. A functionally intact clearance pathway is thus essential for rapid and efficient elimination and detoxification of free Hb and prevention of its deleterious effects [2, 3].

The CD163 receptor facilitates endocytosis of free Hb and Hb-haptoglobin (Hb:Hp) complexes for intralysosomal processing by blood monocytes and resident tissue macrophages, primarily in the liver and spleen [4–6]. When eventually delivered to the cytoplasm, the globin-free heme is degraded by heme-oxygenase-1 (HO-1) [7, 8]. A diminished pool of monocytes/macrophages, as well as any loss of lysosomal function or lack of HO-1 activity, may therefore compromise physiologic Hb detoxification, increasing the likelihood of pathology [9, 10].

Chloroquine is a lysosomotropic weak base and accumulates within acidic cellular compartments. The pharmacologic action of chloroquine includes an increase in intralysosomal pH, preventing fusion of endosomes and lysosomes, and, consequently, disruption of intracellular trafficking [11–13]. Historically, this agent was widely used for the treatment of malaria—a prototypic hemolytic condition. The efficacy of chloroquine as an antimalarial drug is owed to inhibition of heme catabolism in plasmodium parasites. By blocking polymerization of Hb-derived ferriprotoporphyrin IX, highly toxic heme-chloroquine complexes accumulate, thus limiting parasite survival [14]. In contrast, preserving the Hb clearance pathway in malaria infected patients is critical. Oxidative heme toxicity to the blood-brain barrier has been intimately linked to some of the most severe cerebral complications of this disease [15], and efficient Hb-iron recycling is critical to support erythropoiesis during severe anemia, which is one of the major worldwide causes of malaria death [16–18]. It is so far not known whether chloroquine could impair the Hb clearance pathway of human macrophages. 

Although chloroquine has been largely abandoned as an antimalarial agent due to widespread emergence of resistant parasitic strains and availability of alternative medications [19, 20], clinical interest was regained in recent years, based on its utility as an effective immunomodulator. Chloroquine and its hydroxyl derivative, hydroxychloroquine, are now widely used as adjuncts in treatment of autoimmune diseases [21, 22]. However, hemolytic anemia is a notable and frequent manifestation in autoimmunity, such as in patients with systemic lupus erythematodes (SLEs). The potential drawbacks of chloroquine therapy, relative to impeded Hb detoxification, remain unknown and could overshadow the protective immunomodulatory benefits in some patients with a significant hemolytic disease component. 

For the present study, we developed a new mass-spectrometry-based quantification method to track directly CD163 mediated uptake of Hb:Hp into lysosomes and subsequent decay processes. We found that chloroquine treatment resulted in intracellular Hb trapping, abolished HO-1 expression, and suppression of the adaptive iron metabolism response. Our results suggest that chloroquine interferes with the hemoglobin scavenger pathway, potentially compromising efficient Hb clearance and aggravating ill effects of extracellular Hb.

## 2. Results

### 2.1. Quantification of Internalized Hb Peptides Using Single Reaction Monitoring (SRM)

We developed a protein-targeted single reaction monitoring (SRM) method for quantifying cell associated Hb by mass-spectrometry. The method is based on the sequence-specific identification and label-free quantification of paired peptide precursor- and fragment-ion masses, also known as transitions. Definitive transitions to be quantified were selected according to *in silico* peptide characteristics, inspection of relative peak intensities of different transitions, and retention time alignment of SRM signals. As such, we monitored three Hb peptides with at least three fragment ions per peptide. Optimal SRM signals were obtained for the following three Hb peptides: SAVTALWGK (*m/z *466.76), MFLSFPTTK (*m/z* 536.27), and LLVVYPWTQR (*m/z* 637.83). 

**Figure 1:**
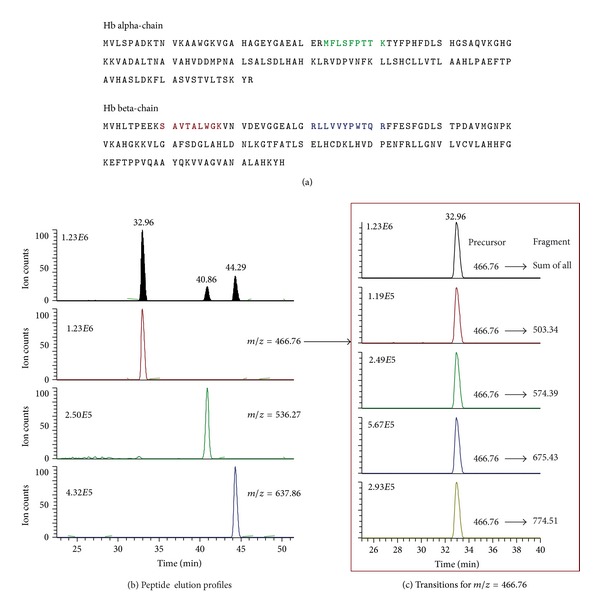
SRM of Hb peptides. (a) Amino acid sequences of the alpha and beta chains of Hb. The selected peptides are indicated as bold text (one peptide in the alpha chain, two peptides in the beta chain). (b) Elution chromatography profiles of the three Hb peptides (SAVTALWGK, MFLSFPTTK, and LLVVYPWTQR). (c) Transition profiles of precursor and fragment ions of SAVTALWGK peptide coeluting at 32.96 min.

In Figure 1, representative ion chromatograms of precursors and fragment ions are shown. Note that fragment ions of the same precursor peptide coelute at the same retention time. The relative precursor-ion quantity was calculated as the sum of the peak areas of all related fragment-ion chromatograms and was assumed to correlate with the respective Hb peptide quantity being present in the endosomal-lysosomal compartment. 

### 2.2. Chloroquine Promotes Intracellular Hb Accumulation

**Figure 2:**
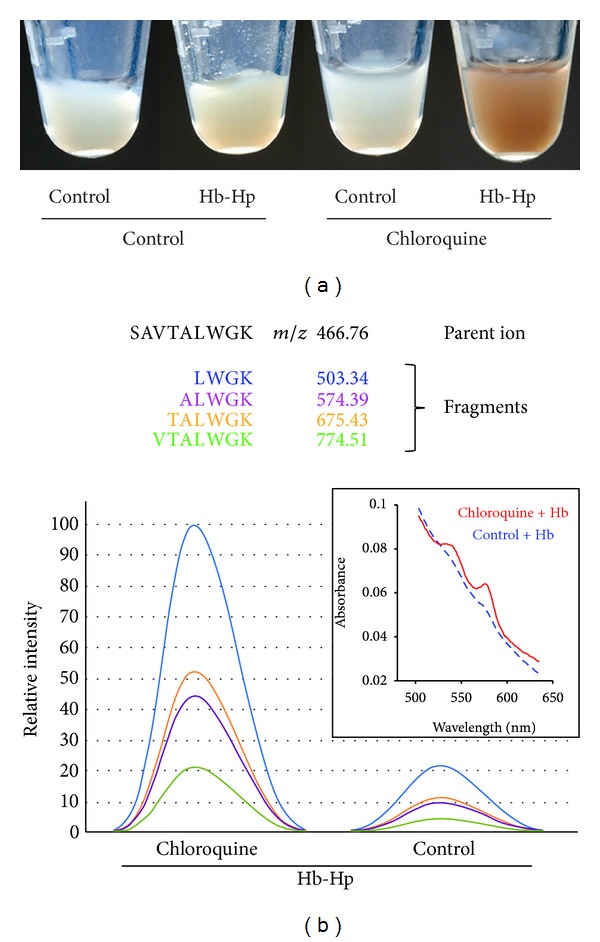
Chloroquine treatment causes intracellular accumulation of heme and globin. (a) CD163-positive HEK cells were exposed to 2 mg/mL Hb:Hp for 12 hours in the presence or absence of chloroquine (10 *μ*M). The image shows pellets of extensively washed cells at the end of the treatment period. Chloroquine-treated cells appear intensely red-brown after Hb:Hp exposure, indicating high intracellular heme. (b) Integration of fragment-ion signal intensities for the Hb β-chain specific peptide SAVTALWGK, as detected by SRM. Sequences and *m/z* ratios of the targeted peptide fragments are indicated. The integrated signals confirm that the Hb peptide accumulates in the chloroquine-treated cells compared to control cells. The insert shows VIS spectra in the range of 500 nm–650 nm of cell lysates, showing a characteristic Hb absorption component in the chloroquine + Hb treated cells.

Figure 2(a) shows representative CD163-transduced HEK-293 cell pellets obtained after exposure of the cells to Hb:Hp (2 mg/mL) for 12 hours. We have previously reported that the CD163-HEK-293 cell is a valuable model to study Hb endocytosis and subsequent cellular responses *in vitro *[5, 7]. CD163-HEK-293 cells treated with chloroquine (10 *μ*M) 30 min before and during Hb:Hp exposure clearly differed in appearance from control cells. The red-brown color of chloroquine-treated cells indicated greater accumulation of heme, as opposed to those not pretreated. This impression could be substantiated by spectrophotometric analysis of the lysed cell pellets which shows a significant Hb absorbance component in chloroquine-treated cells with characteristic absorbance peaks at 540 nm and 575 nm but much less than in control cells (insert in Figure 2(b)). In addition a significant absorption peak was detected at 630 nm, which suggests that a fraction of Hb is oxidized to metHb (Fe^3+^) after endocytosis within the acidic and proteolytic endolysosomal compartment (not shown). In Figure 2(b), the relative intensity profiles of SRM-trapped fragment ions is depicted for one representative Hb peptide. Compared with control samples, this analysis indicates a higher globin protein abundance in the Hb *plus* chloroquine-treated CD163-HEK-293 cell lysates compared to cells that were exposed to Hb in the absence of chloroquine. Collectively, the increased SRM signal in parallel to the brown appearance of the cell pellets confirms that not only heme but also the protein component (globin) of Hb massively accumulates in the chloroquine-treated cells.

### 2.3. Chloroquine Limits Intracellular Hb Clearance without Altering Cellular Uptake Capacity

**Figure 3:**
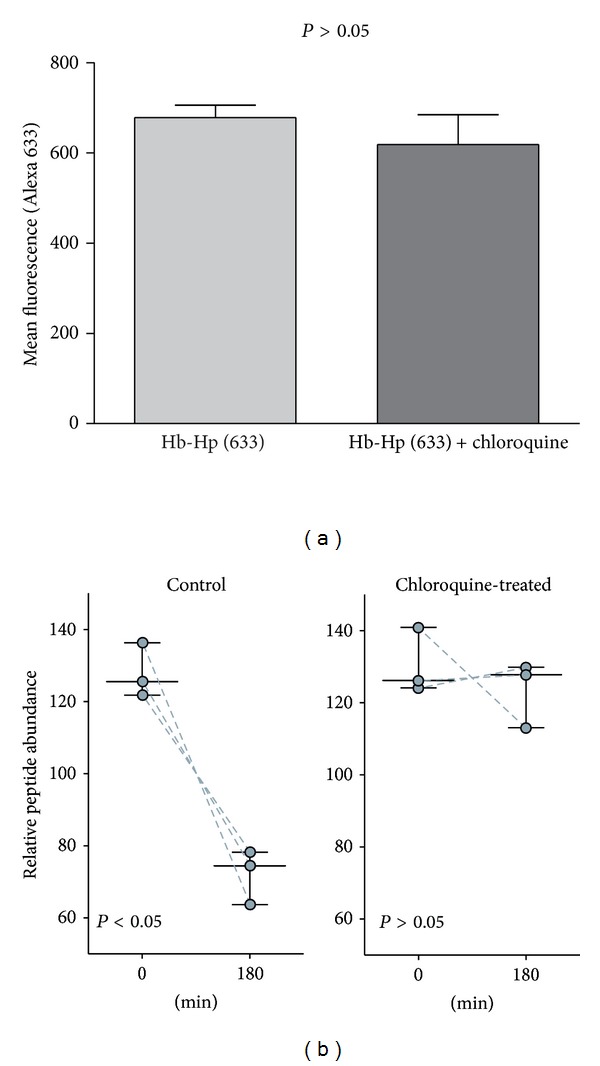
Chloroquine impairs Hb degradation but not primary cellular Hb uptake. (a) Fluorescent Hb:Hp (Alexa 633) uptake capacity of chloroquine pretreated (30 min) and control CD163-positive HEK cells (values given as mean ± SEM of channel fluorescence from three independent experiments). No statistical difference was observed (*P* > 0.05). (b) CD163-positive HEK cells, with and without chloroquine pretreatment (as shown in Figure 2), were incubated for 12 hours with Hb:Hp 2 mg/mL. After removing Hb from culture medium by extensive washing, the cell samples were either immediately lysed or further incubated for 180 min before intracellular Hb-specific peptides were relatively quantified by SRM. The following three Hb peptides were monitored and are individually represented in the graph: SAVTALGK, MFLSFPTTK, and LLVVYPWTQR (see Figure 1). The Hb decay is significantly less in the chloroquine-treated cells (control 0 versus 180 min *P* < 0.05, chloroquine-treated 0 versus 180 min *P* > 0.05). Data points represent mean peptide abundance values that were determined in three independent experiments.

We further addressed the question of whether chloroquine modifies CD163 mediated Hb uptake capacity or whether it interferes with the subsequent intracellular degradation pathway of Hb. The fluorescence Hb:Hp uptake assay shown in Figure 3(a) shows that the uptake capacity of the chloroquine-treated cells within the first 30 min of incubation is equal to control cells, indicating that the accumulation of Hb observed in chloroquine-treated cells must be the result of a diminished Hb degradation, rather than increased Hb uptake.  Figure 3(b) shows the SRM-determined decay of the endocytosed Hb peptides in chloroquine and control cell populations. After incubation with Hb:Hp (2 mg/mL for 12 hours), the cells were washed with phosphate-buffered saline (PBS) containing EDTA to remove noninternalized Hb. Cell samples were either lyzed immediately after washing (0-hour sample) or further incubated at 37°C and lysed after 180 minutes to quantify intracellular Hb peptides relative to the 0-hour samples. While we observed a considerable decrease of Hb peptides in control cells, Hb elimination was blocked by chloroquine treatment. Therefore, our results suggest that chloroquine impairs elimination of Hb by interfering with lysosomal Hb degradation.

### 2.4. Chloroquine Inhibits HO-1 Expression and Creates a State of Relative Intracellular Iron Deprivation

**Figure 4:**
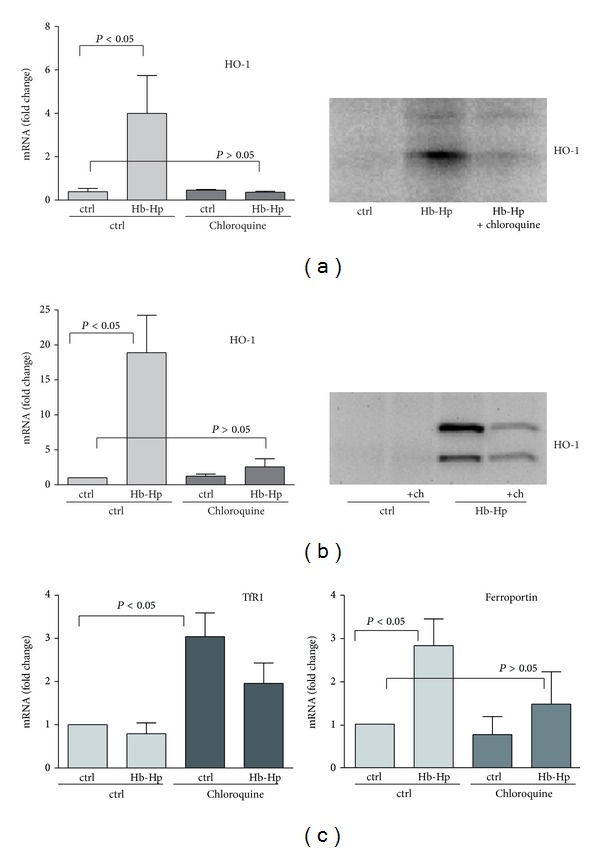
Chloroquine treatment attenuates the cellular HO-1 and iron metabolism response to Hb:Hp exposure. (a) Relative mRNA (left) and protein (right, Western blot) expression levels of HO-1 in chloroquine-treated and untreated CD163-positive HEK cells after stimulation with Hb:Hp (2 mg/mL) for 8 hours (values represent mean ± SEM of at least three independent experiments). (b) Relative mRNA (left) and protein (right, Western blot) expression levels of HO-1 in human monocyte derived macrophages after Hb:Hp (2 mg/mL) exposure for 8 hours in the presence or absence of chloroquine. (c) Relative changes of transferrin receptor (TfR1) and ferroportin mRNA expression in human monocyte derived macrophages after Hb:Hp (2 mg/mL) exposure for 8 hours in the presence or absence of chloroquine. All mRNA changes are given relative to control cells (data indicate mean ± SEM of at least three independent experiments).

Heme detoxification relies primarily on inducible HO-1 enzyme, which enables degradation of Hb to iron, bilirubin, and CO [23, 24]. With addition of chloroquine, the strong induction of HO-1 expression, that is, normally observed upon Hb:Hp exposure of CD163-HEK-293 cells, is suppressed at the mRNA and protein levels (Figure 4(a)). We confirmed this chloroquine effect also in human peripheral blood monocyte derived macrophages (Figure 4(b)). Also in this cell type, which represents the natural Hb clearance compartment in human, HO-1 induction by Hb:Hp was suppressed by chloroquine.

By limiting Hb:Hp triggered HO-1 expression and cellular heme metabolism, chloroquine could profoundly modify macrophage iron homeostasis. We found that exposure to chloroquine in the absence of Hb stimulated mRNA induction of the transferrin receptor (the principal iron importer) (Figure 4(c)). This implies that under the pharmacologic influence of chloroquine intracellular levels of free iron are shifted towards a state of relative iron deprivation. Accordingly, while Hb:Hp uptake stimulated mRNA induction of the principal iron exporter ferroportin in untreated macrophages, chloroquine treatment blunts this adaptive response, presumably by limiting iron recovery from Hb (Figure 4(c)). Collectively, these data support our hypothesis that chloroquine could limit the capacity of macrophages to detoxify heme and to recycle iron.

### 2.5. Chloroquine Paralyzes Endocytic Hb:Hp Trafficking

**Figure 5:**
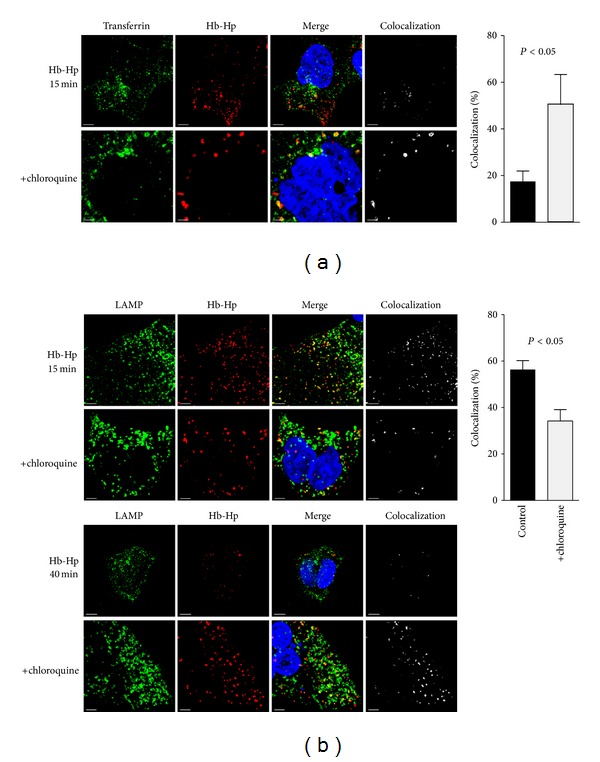
Chloroquine impairs Hb:Hp trafficking through the endosomal-lysosomal compartment. (a) CD163-positive HEK cells incubated 15 min after endocytosis with Alexa 594-labeled Hb:Hp complexes and Alexa 488-labeled transferrin, with and without chloroquine pretreatment. The proportion of endocytosed Hb:Hp complexes colocalizing with the early endosomal compartment (transferrin-positive) increases substantially after chloroquine exposure. (b) CD163-positive HEK cells incubated for 15 min (upper two rows) or 40 min (lower two rows) after endocytosis with Alexa 594-labeled Hb:Hp complexes. The lysosomal compartment is stained with anti-LAMP-1 antibody and Alexa 488-labeled secondary antibody. Early colocalization of Hb:Hp complexes in the lysosomal (LAMP-1) compartment after 15 min is considerably reduced by pretreatment with chloroquine. In contrast, after 40 min, chloroquine pretreatment leads to marked accumulation of Hb:Hp complexes in the lysosomes. Optical magnification of all images is 630x. DAPI-stained nuclei are blue, and scale bars are spaced at 5 *μ*m. The percentage of colocalization is shown in the graphs on the right with black bars indicating control cells and gray bars indicating chloroquine-treated cells; *n* = 4-5 images per condition that were recorded on independent samples.

As a lysosomotropic agent, chloroquine is likely to alter the dynamics of Hb:Hp trafficking though the endosomal-lysosomal compartments. We performed confocal fluorescence microscopy to examine how chloroquine changes the temporal and spatial relationships of endocytosed Hb:Hp with the early endosomal marker transferrin and the lysosome-associated-membrane-protein-1 (LAMP-1). After 15 min (Figure 5(a)), we found that chloroquine favoured colocalization of endocytosed Hb:Hp with the early endosomal marker transferrin, while more Hb:Hp was already transported from early endosomes to LAMP-1-positive lysosomes in untreated cells (Figure 5(b)). Only in the later decay phase, after 40 min, Hb:Hp was found to predominantly colocalize with lysosomal LAMP-1 in chloroquine-treated cells, whereas, in the absence of chloroquine, Hb:Hp was largely degraded at this later time point. Both early endosomes and lysosomes showed uniform enlargement under chloroquine.

These data support the contention that chloroquine interferes significantly with endocytic Hb:Hp trafficking and degradation, presumably reducing heme access to cytoplasmic HO-1.

## 3. Discussion

Our data provide evidence that chloroquine or its derivatives may interfere with an established pathway for Hb clearance, thereby inhibiting heme detoxification and, potentially, heme-iron recycling. Specifically, we found that chloroquine blocks Hb:Hp degradation by paralyzing lysosomal function and limiting heme access to HO-1, the primary enzyme of Hb-heme catabolism. 

For this study, we developed a method to track intracellular globin concentration after endocytosis of Hb:Hp complexes. Although assays by spectrophotometry might enable Hb quantification in cells, based on the characteristic absorption spectra of its heme moiety, quantification of the protein itself remains challenging. Flow-cytometry assays, as alternative methods, require fluorescent tracers, which may alter the natural elimination rate of a target protein or metabolically skew a protein of interest. Furthermore, the elimination of the fluorescent tracer may disconnect from heme/Hb elimination in the acidic and proteolytic environment of the lysosomes. 

Selected reaction monitoring (SRM) represents a powerful and sensitive alternative method that allows direct and sequence-specific protein measurement at very low-concentration. Therefore, we developed an SRM based method to determine relative intracellular Hb concentrations and track the decay rate of Hb-specific peptides after endocytosis. Our SRM method may be of use in other experimental settings to evaluate hemolytic processes, tissue distribution of free Hb, and Hb metabolism in various disease states. Furthermore, similar assays may be developed to monitor dynamic trafficking and clearance processes of other proteins. Similar methods have been used previously to quantitatively study dynamic interaction of endocytic receptors with protein ligands and to investigate protein catabolic processes [25, 26].

Under our experimental conditions, which involved drug concentrations within the range of peak plasma concentrations measured in chloroquine-treated patients [27, 28], the agent does not impair initial endocytosis of Hb:Hp complexes by the CD163 scavenger receptor. However, chloroquine treatment drastically slows the decay rate of Hb. The resulting massive Hb accumulation is attributable to the known lysosomotropic action of this agent. Apparently, degradation of Hb requires an intact lysosomal system for cytoplasmic delivery of Hb-derived heme and further catabolism by HO-1. Morphologically, the result of deregulated Hb trafficking becomes apparent as a global congestive enlargement of lysosomes that we observed by confocal microscopy. In chloroquine-treated cells the Hb:Hp complexes are stacked within the early endosomal compartment, reaching the lysosome compartment only 40-minutes after completed endocytosis. Normally, trafficking of Hb:Hp complexes through the endosomal-lysosomal compartment is largely completed at this time point.

We have previously shown that Hb-heme internalization by CD163 induces high levels of HO-1 mRNA and protein expression [7, 29]. Upon treatment with chloroquine, Hb is still internalized into the endocytic compartment by CD163 receptor, but the HO-1 expression is abolished, possibly due to limited delivery of Hb-derived heme into the cytoplasm. HO-1 is pivotal in preventing Hb toxicity by catabolizing heme to the anti-inflammatory and antioxidant byproducts: bilirubin, carbon monoxide, and ferritin [23, 24]. However, if this protective mechanism is suppressed, susceptibility to the oxidative and proinflammatory toxicities of extracellular Hb may increase, particularly during acute hemolytic episodes.

It could be hypothesized that inhibition of Hb detoxification by chloroquine treatment could be particularly deleterious in patients infected with chloroquine resistant plasmodium parasites. The 1.63-fold increased risk of cerebral malaria and 4-fold increased risk of mortality in chloroquine-treated children that was observed in a study of home-treatment strategies for febrile children in Nigeria could endorse this hypothesis [30].

We also found that chloroquine adversely modifies Hb:Hp triggered adaptation of iron homeostasis in human macrophages preventing release of heme iron into the recycle pathway. Under normal conditions, the heme-iron load, which is imparted by endocytosis of Hb:Hp complexes, causes a rapid downregulation of the transferrin receptor and a concurrent induction of the iron exporter ferroportin [8]. This adaptive response assures that iron homeostasis is shifted towards enhanced iron supply to the bone marrow, which is a critical process in hemolytic anemia [31]. Chloroquine treatment appears to suppress this adaptive gene regulation and may therefore impair heme-iron recycling.

In conclusion, our studies indicate that chloroquine interferes with the detoxification pathway of extracellular Hb. In some conditions chloroquine treatment may therefore aggravate the deleterious effects of hemolysis, limiting erythropoietic heme-iron supply and promoting inflammation and tissue damage in patients with malaria or autoimmune hemolytic anemia.

## 4. Material and Methods

### 4.1. HEK Cells Transfection and Culture

Human-embryonic-kidney- (HEK-) 293 cells (Invitrogen, Basel, Switzerland) were cultured in DMEM (Invitrogen) containing 10% FCS (Invitrogen). CD163-expressing lentiviral-transduced HEK-293 cells were generated as described elsewhere [5] and maintained with 5 *μ*g/mL blasticidin (Invitrogen), which was removed from cultures at least one cell passage prior to experiments. 

### 4.2. Macrophage Isolation

Human-blood-derived monocytes were prepared from buffy coats of healthy donors, as purchased from the Swiss Red Cross Blood Bank (Swiss Red Cross) and already described [32]. These cells were cultured in Iscove modified Dulbecco medium (Invitrogen), supplemented by 10% heat-inactivated, pooled human serum having negligible residual free Hb by design. Written informed consent was obtained from all donors, in accordance with the Declaration of Helsinki, and all the work was approved by the ethics review board of the Canton of Zurich. 

### 4.3. Proteomic Sample Preparation and TSQ Mass-Spectrometry

Total cellular protein was extracted using CelLytic-M reagent (Sigma-Aldrich) supplemented by Complete Mini Protease Inhibitor (Roche Diagnostics). After three freeze-thaw cycles, cellular debris was removed by a 30-minute centrifugation (16000 ×g).

The protein concentration of each sample was determined using Bradford assay (Bio-Rad, Hercules, CA, USA). Samples were precipitated by adding TCA (10% final concentration) and incubated on ice for 20 min. Following centrifugation (14000 ×g), the supernatant was removed, and the pellets were triple-washed in ice-cold acetone. Afterward, they were dried 5 min at 95°C and reconstituted in 0.1% RapiGest (Waters). Disulfide bonds were reduced by DTT additive and finally blocked by treatment with iodoacetamide. The pellets were next subjected to overnight digestion by trypsin (1 : 10 ratio, trypsin to protein) at 37°C. Lyophilized peptide mixtures were ultimately dissolved in 5% ACN and 0.1% formic acid for loading onto a 5 *μ*m C18 column (PicoFrit Column Hyp. Gold, AQ 5 *μ*m, 75 *μ*m  × 100 mm, New Objective Inc.). 

A TSQ Vantage Triple Quadrupole mass spectrometer (Thermo Fisher Scientific, Waltham, MA, USA), equipped with a nanoelectrospray ionization source, was used for SRM measurements. Chromatographic peptide separations were performed on an Eksigent 1D Nano-LC system (Eksigent Technologies), applying a 55 min gradient from 5 to 50% v/v acetonitrile in water, both amended with 0.1% v/v formic acid. The LC operational flow rate was 500 nL min^−1^. Mass-spectrometry was done in SRM mode; Q1 and Q3 were set at resolutions 0.2 and 0.7 Da, respectively. A spray voltage of +1400 V was used with a heated ion transfer capillary setting of 120°C for desolvation. A 20 ms dwell time was set, with 0.002 *m/z* scan width. 

### 4.4. Analysis by Selected Reaction Monitoring

SRM methods were developed and optimized as described elsewhere [33–35]. Tryptic peptides lacking methionine or cysteine were measured, and transitions (for three peptides per protein) were selected considering first maximum signal intensities observed during LC-MS/MS, with further refinement with respect to signal-to-noise measurements during the SRM trials. SRM peak areas were calculated by using Pinpoint software (Thermo Fisher Scientific, Waltham, MA, USA). All SRM measurements were performed on three biologic replicates. 

### 4.5. Quantitative Real-Time Reverse Transcription-Polymerase Chain Reaction (RT-PCR)

Real-time RT-PCR was performed on a Fast Real-Time PCR System Instrument (Applied Biosystems) using TaqMan reverse transcription and SYBR Green master mix PCR reagents (Applied Biosystems) as described. Gene-specific quantitative data were corrected for *HPRT* mRNA abundance in the respective samples and are expressed as fold expression relative to the control. 

### 4.6. Western Blot Analysis

Western blots were performed using standard techniques and a single primary HO-1 antibody (SPA-896; Stressgen). Sample loading was adjusted for equal protein per lane after measurement of the protein concentration by Bradford assay (Bio-Rad, Hercules, CA, USA). The horseradish peroxidase-conjugated secondary antibodies (Amersham Biosciences) used for detection were diluted 1 : 10000. Blots were developed with ECL Plus Western blotting detection reagent (Amersham Biosciences) and analyzed on a ChemiDoc XRS system with Quantity One 1-D Analysis software, version 4.5.0 (Bio-Rad). 

### 4.7. Analysis of Fluorescent Hb:Hp Uptake

CD163-transduced HEK-293 were incubated for 30 min at 37°C with Alexa 633-labeled Hb:Hp (3 *μ*g/mL) [32]. The cells were washed with EDTA containing buffer and resuspended in phosphate-buffered saline (PBS) for analysis by a FACSCalibur (Becton Dickinson). Data were analyzed with CellQuest (Becton Dickinson). 

### 4.8. Confocal Fluorescence Microscopy

For fluorescence microscopy, HEK cells were cultured on baked (4 h at 220°C), endotoxin-free, sterile, round, 12 mm glass coverslips (Hecht-Assistent, Germany), placed in 24-cluster wells, and pretreated 2 h before examination with 6.25 mcg/cm^2^ poly-D-lysine hydrobromide (BD Biosciences, San Jose, CA, USA). Pretreatment with chloroquine (10 *μ*M) lasted 12 h. Fluorescent-labeled Hb:Hp complexes (with or without fluorescent transferrin) required a 15 min incubation in cell culture medium containing 10% FCS. To determine subcellular localization of Hb:Hp 15 or 40 min after endocytosis, the medium was changed, and a second incubation (with or without transferrin) followed in the absence of fluorescent Hb:Hp complexes. Labeling of Hp (Sigma Chemical Co.) was done using Alexa 594 protein-labeling kit (Molecular Probes, Eugene, OR, USA). Hb:Hp complexes were generated by combining Hb and fluorescent Hp at a 1 : 1 molar ratio, 10 min before examination. Hb:Hp complexes were employed at 10 *μ*g/mL concentration. Transferrin was labeled using the Alexa 488 protein-labeling kit and employed at 20 *μ*g/mL concentration. Finally, the cells were triple-washed with PBS, pH 7.4 (Sigma Chemical Co., St. Louis, MO, USA), fixed 15 min in PBS with 2.5% paraformaldehyde, and permeabilized for 5 min at room temperature in 0.1% Triton X-100 (Sigma Chemical Co.) and PBS. After washing, nonspecific binding sites were blocked for 1 h in 10% goat serum and PBS, supplemented with 1% bovine serum albumin ((BSA) Sigma Chemical Co.), again at room temperature. 

Staining of LAMP-1 was accomplished by incubation 1 h at room temperature with mouse anti-LAMP-1 antibody (RDI Research Diagnostics, Concord, MA, USA), diluted 1 : 800 in PBS supplemented by 1% goat serum and 0.1% BSA. Alexa Fluor 488 goat anti-mouse IgG (Molecular Probes) served as a secondary antibody, using 10 *μ*g/mL 4,6-diamidino-2-phenylindole ((DAPI) Sigma Chemical Co.) as a nuclear counterstain. After three washes in PBS, coverslips were applied using ProLong Gold antifade reagent-mounting medium (Molecular Probes). Optical sections at 0.125 *μ*m were viewed under a Leica SP5 confocal laser-scanning microscope (Leica, Heidelberg, Germany), using default magnification of 630x. Images were imported into Imaris software, version 6.1.2 (Bitplane Scientific Software, Zurich, Switzerland).

The extent of colocalization was quantified on the basis of intensity per colocalization module in Imaris. To differentiate fluorescent Hb:Hp (red channel) colocalization with transferrin or LAMP-1 (both green channel), the red channel contribution to colocalized areas was analyzed using a fixed threshold of 127.5 in both channel histograms. 

### 4.9. Statistical Analysis

Data were analyzed with GraphPad Prism version 4.0. We used an unpaired 2-tailed Student's *t*-test for comparisons of groups, with ANOVA where applicable. *P* values <0.05 were considered statistically significant. 

## Abbreviations

Hb: Hemoglobin
Hp: Haptoglobin
HO-1: Heme-oxygenase-1
GAPDH: Glyceraldehyde-3-phosphate dehydrogenase
LAMP-1: Lysosomal-associated-membrane-protein-1
SRM: Single reaction monitoring
ACN: Acetonitrile.
